# Long-Acting Reversible Contraception Uptake and Associated Factors among Women of Reproductive Age in Rural Kenya

**DOI:** 10.3390/ijerph16091543

**Published:** 2019-05-01

**Authors:** Susan Ontiri, Gathari Ndirangu, Mark Kabue, Regien Biesma, Jelle Stekelenburg, Collins Ouma

**Affiliations:** 1 Jhpiego Corporation, An Affiliate of Johns Hopkins University, Nairobi 00800, Kenya; gathari.ndirangu@jhpiego.org; 2 Jhpiego Corporation, An Affiliate of Johns Hopkins University, Baltimore, MD 21231, USA; Mark.kabue@jhpiego.org; 3 Department of Health Sciences, Global Health, University Medical Centre Groningen/University of Groningen, 9713 GZ Groningen, The Netherlands; r.biesma@umcg.nl (R.B.); jelle.stekelenburg@online.nl (J.S.); 4 Department of Obstetrics & Gynecology, Leeuwarden Medical Centre, 8934 AD Leeuwarden, The Netherlands; 5 Department of Biomedical Sciences and Technology, Maseno University, Private Bag, Maseno 40105, Kenya; collinouma@yahoo.com

**Keywords:** family planning, long-acting reversible contraception, factors, uptake, discontinuation

## Abstract

In the last two decades, the use of short-acting methods of contraception has driven the increase of contraceptive use in Kenya. We assessed the factors associated with uptake of long-acting reversible contraception by women seeking family planning services in public health facilities in Kakamega County, Kenya. A mixed methods cross-sectional study through client exit surveys among 423 women seeking family planning services was done at 12 public health facilities in Kakamega County. Twelve in-depth interviews with health care providers from the study facilities further explored practices in provision of long-acting reversible contraception (LARC). Among women initiating contraceptive use, LARC method utilization was 20.6%. Women’s tertiary education level, Protestant Christian religion, age at first birth, and having no desire for more children were significantly associated with utilization of LARC. Structural factors including shortage of human resource, provider bias and lack of adequate skills on provision of services were identified as key barriers to uptake of long-acting reversible contraception services.

## 1. Introduction

Unintended pregnancy is a major problem among sexually active women and can result from incorrect, inconsistent, or non-use of contraception, or contraceptive failure—that is, becoming pregnant while using a family planning (FP) method. Long-acting reversible contraception (LARC), which includes intrauterine devices (IUDs) and sub-dermal implants, have many desirable attributes, such as highly effective protection against unwanted pregnancies and few contraindications. They do not require the users’ ongoing effort for long-term and effective use following initial insertion, are cost-effective, do not require frequent visits for resupply and are reversible with a rapid return to fertility after removal [1]. The World Health Organization (WHO) estimates that only one unintended pregnancy occurs among every 2000-implant users in the first year of use; the effectiveness of IUDs are nearly the same [2]. In contrast, failure rates in the first year of typical use for popular short-term methods are considerably higher: 90 unintended pregnancies per 1000 users of pills, and 60 unintended pregnancies per 1000 users of the depo medroxyprogesterone acetate (DMPA) injectable, which contains progestogen hormone. Thus, implants and IUDs are 120 times more effective than the injectable and 180 times more effective than the pill [3]. Other studies indicate that nearly a fifth of unintended conceptions are among women who use a modern, short-term contraceptive, mostly due to poor adherence [4].

LARC methods that are widely available in Kenya include the two-rod levonorgestrel, the one-rod etonogestrel implant, and the copper IUD. Kenya has made tremendous progress in increasing uptake of contraceptive use over the years. As per the Kenya Demographic Health Survey, use of modern methods in Kenya has increased from 32% in 2003 to 53% in 2014 among married women aged 15–49 years, which has largely been driven by use of short-term methods. Uptake of LARC has steadily increased from 4.1% in 2003 to 13.3% in 2014 [5]. The more recent findings of Performance Monitoring and Accountability (PMA) 2020 round 7 data (November–December 2018) for Kenya, indicates a modern contraceptive prevalence rate (mCPR) of 60.7% with a substantial improvement in long acting and permanent methods (LAPM) use, currently at 28.5% [6]. Despite the progress Kenya has made in contraceptive use over the last four decades, discontinuation rates are still high, with one out of three women discontinuing use by 12 months. Discontinuation rates are higher for common short-term methods (45% for pills, 31% for injectables, and 43% for male condoms) compared with LARC (6% for IUDs, 8% for implants) [5]. Meanwhile, LARC users are less likely to discontinue use due to side effects and health concerns (4% of IUD users, 7% of implant users) compared with common short-acting, hormonal methods (16% of pill users, 14% of injectable users). Thus, a shift to long-acting methods is likely to reduce discontinuation rates and may better meet women’s needs.

Several studies have shown that socio-demographic factors such as age of women, marital status, education level [7,8], place of residence [9], and religion [10] influence LARC uptake in different settings. Many studies have also cited reproductive health factors such as fertility intention, parity, and desire for FP as influencers of uptake of LARC methods and contraceptive use in general. In other studies, parity [11,12], desired family size, women who gave birth prematurely, previous history of abortion [13], women who had ever experienced an unwanted pregnancy [14], and women who had visited a clinic in the past year for FP services [15] were identified as reproductive health factors associated with LARC use. While many studies on LARC have been carried out to prove the effectiveness of the methods, the literature regarding determinants of LARC uptake among contraceptive users have focused mainly on certain women population sub-sets, such as postpartum women and adolescents.

The current study assessed factors associated with LARC uptake among women of reproductive age, 15 to 49 years, and evaluated socio-demographic and reproductive factors associated with the use of LARC methods in Kakamega, a rural county in Kenya. Understanding the factors associated with LARC uptake in a rural setting provides information that can help policy makers adjust their programs to further increase utilization of these methods that are more effective in reducing unplanned pregnancies.

## 2. Materials and Methods

### 2.1. Study Setting

We implemented the study in 12 public health facilities in Kakamega County, in Western Kenya, between August and September 2015. Kakamega County is rural county as classified by the Kenya National Bureau of Statistics. It was selected because it is the second most populous county in Kenya (and the most populous rural county) with a population of 1,660,051 per the 2009 population census data. Women in the reproductive age group formed 35% of the county’s population and the fertility rate, at 4.4, is higher than the national average of 3.9 children per woman. The increase in growth of FP use is plateauing, as per the Track20 family planning S-Curve [16], with short-term methods driving the high contraceptive prevalence rate of 62.1% [5].

### 2.2. Study Design

The study team used a cross-sectional design. Public health facilities were selected through multistage and probability proportional to size methods.

### 2.3. Study Population

The study population were women of reproductive age (15–49 years) residing in Kakamega County for the last six months prior to the survey, and visiting the FP clinic for initiation of contraception services. The study excluded women who were coming for contraceptive removal services. The study adopted a client exit interview approach for data collection at the FP clinic.

### 2.4. Sampling Procedure and Sample Size

We calculated a sample size of 423 participants using the single sample proportion formula by considering 50% proportion of LARC method selectors with a 95% confidence level, 5% margin of error, and 10% non-response rate. At the time of study design, no published literature on the prevalence of LARC uptake in the public health facilities of Kakamega County was available, hence the choice of 50% as the proportion of LARC method users. This estimated prevalence was used on assumption that Kakamega is a populous county with unknown variability in the proportion of LARC uptake at public health facilities [17]. A multistage stratified sampling technique was used to ensure homogenous and representative sampling in the population. Only public health facilities were included in the study because of the uniformity in provision of free cost FP services. From each of Kakamega’s 12 sub-counties, one facility was purposively selected during the first stage of sampling, based on the highest catchment area that the facility serves per the Ministry of Health records. Thus, a total of 12 health facilities that ranged from level 4 (referral hospital) to level 3 (primary health care facility) were sampled. The second stage of sampling was at the FP clinic, where systematic random sampling was used to select the clients to be interviewed. The sample size for each facility was determined based on probability proportional to size using the catchment area population. At the facility level, the sampling interval of individual participants was calculated based on the daily FP caseload and the facility sample size. In addition, in-depth interviews were conducted with 12 FP providers, one in each of the sampled facilities.

### 2.5. Ethical Clearance

Maseno University Ethical Review Committee granted ethical approval under reference number MSU/DRP/MUERC/00177/15.

### 2.6. Data Collection Procedures

The 12 sampled facilities had health care workers who were skilled in the provision of all contraceptive methods with the exception of permanent methods, which were only provided in the county referral hospital. Data collection took place during FP clinic operational hours which is between 9 a.m. and 12 p.m., Monday to Friday. Quantitative data were collected through face-to-face interviews using a structured and pre-tested questionnaire. The questionnaires were first prepared in English, translated into Swahili and then back-translated to English to maintain consistency. Twelve research assistants who had a diploma collected the data under the supervision of two study coordinators with a graduate degree. To assure for data quality, the tool was pre-tested on 10% of the actual sample size in the neighboring county and training was given to the research assistants. The research assistants were stationed at the facility exit and approached women to participate in the study. A total of 867 women were approached, of whom 423 met the inclusion criteria. The research assistants consented the women and then administered the survey, which lasted approximately 20 min. Systematic random sampling was used to select the clients to be interviewed. The sampling interval was calculated for each facility based on the average daily FP caseload and the facility sample size. We conducted data collection for 8 days, interviewing 5 to 10 clients per facility daily until the targeted sample was achieved. Paper questionnaires were checked for completeness and consistency daily by the supervisor. For the qualitative data, three trained qualitative researchers conducted in-depth interviews with the 12 providers and took notes.

The main outcome of interest in this study was LARC uptake; implants and copper IUDs were categorized as LARC, three-month DMPA injectable and oral contraceptive pills were categorized as short-term methods. The independent variables examined in the study were socio-demographic characteristics (age, marital status, educational level, religion, residence and occupation), and reproductive factors (parity, sexual debut age, age at first birth, fertility intention and desired number of children).

### 2.7. Statistical Analysis

#### 2.7.1. Quantitative Data

The data were entered from paper form into Epi-data software and then exported to STATA ver. 13 (StataCorp, College Station, TX, USA) for coding and analysis. The Mann-Whitney U test was used to test for differences in age at first birth and distance to nearest health facility. Bivariate analysis was initially conducted primarily to check for the association between the dependent and independent variables, significant ones (at *p* < 0.05) were then entered into multivariable logistic regression model. All *p* < 0.05 were considered statistically significant.

#### 2.7.2. Qualitative Data

Interviews with health workers occurred in English; data were audio-recorded and transcribed. The English transcripts and available notes were then analyzed manually for content using an emergent approach and latent content analysis. The analyst read through each transcript several times highlighting and labelling blocks of text with related underlying meaning (codes). The identified codes were then subjected to constant comparison before being merged into categories of codes with related meaning. The themes connecting the codes within each category were then identified and are reported descriptively.

## 3. Results

### 3.1. Quantitative Data

#### 3.1.1. Socio-Demographic and Reproductive Characteristics of Study Participants

In this study, 423 women of reproductive age who attended one of the 12 public facilities were interviewed, with a response rate of over 99%. The mean (± standard deviation) age of the participants was 28.3 (±7.3) years; about a third were in the age group between 15–24 years. The majority 306 (72%) were married. In terms of education level, 42% had completed primary formal education and only 3% were illiterate. Most of the respondents 257 (61%) were protestants, the majority 331 (78%) resided in rural areas, and more than half 255 (60%) had some form of occupation. The mean (±standard deviation) distance from the respondent’s residence to a health care facility was 2.5 (±2.3) kilometers. Most (93%) of the respondents had ever given birth, age at first birth was 19.8 (±7.3). More than half 220 (52%) desired three or four children, 27% of the respondents wanted children after 2 years, while a similar proportion (27%) did not want any more children (Table 1).

#### 3.1.2. Contraceptive Method Mix among Women Initiating Use

**Figure 1:**
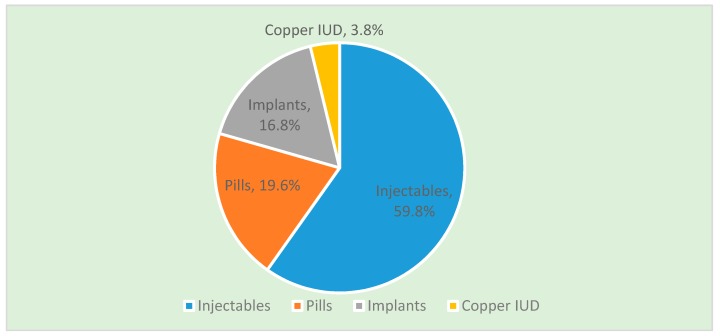
Contraceptive methods chosen by the study participants.

Out of the 423 respondents interviewed, 20.6% (95% CI 17%, 25%) utilized LARC methods while 79.4% (336) chose the short-term methods (Figure 1). The uptake of the different methods decreased in this order: injectables 253 (59.8%), pills 83 (19.6%), implants 71 (16.8%), IUDs 16 (3.8%). During the data collection period, no client opted for voluntary surgical contraception. Some women chose condoms as dual protection method, hence in the analysis, for clients who chose more than one method, the method with the highest effectiveness was considered.

#### 3.1.3. Factors Associated with Uptake of LARC among Women of Reproductive Age

In the bivariate analysis, age, education level, religion, and fertility intention were found to be significantly associated with utilization of LARC (*p* < 0.05). The results from the multivariable logistic regression model (Table 2) revealed that education, religion, and fertility intention were the determinants of LARC uptake. Age at first birth (interquartile range = 3, *p* = 0.025) was also significantly associated with LARC uptake. LARC uptake increased with education level (*p* = 0.029). Women who had tertiary education 30.2% (adjusted odds ratio (aOR) = 2.58, 95% confidence interval (CI) = 1.10–6.03) were more likely to utilize LARC compared to those with primary level or none. Protestant women (16.3%) were less likely to use LARC (aOR = 0.42, 95% CI = 0.24–0.73, *p* = 0.002) compared to Catholics. The strongest predictor of LARC uptake was not desiring more children (31.8%), increasing the likelihood of LARC uptake by almost four times compared to women who desired a child in 2 years (aOR = 3.77; 95% CI = 1.37–10.42, *p* = 0.01). Women who wanted children after 2 years (aOR = 2.94; 95% CI = 1.07–8.08, *p* = 0.037) were also significant and highly likely to use LARC methods compared to those who wanted a child in 2 years.

### 3.2. Qualitative Data

In-depth interview discussions conducted with the 12 health care workers revealed barriers faced by providers in provision of LARC services. Staff shortage was mentioned as a key challenge by all the 12 providers interviewed. In seven out of the 12 sampled facilities, there was only one provider stationed at the FP clinic, and that person was also required to provide services at antenatal and postnatal care, immunization, and child welfare clinics. One provider said: *“A client may come and request for IUD. It takes 20–30 min to do comprehensive counseling, cervical cancer screening, and pelvic examination for pelvic inflammatory diseases to ascertain whether they are fit for the method before you insert the IUD. I advise them to take a short-term method, which takes 5 min to provide, because I have other clients queueing waiting to be served”.*

Eight providers who were interviewed noted lack of skills in providing IUD as a barrier to LARC provision. “I think we as family planning providers contribute to the low uptake of IUD methods because we do not have enough skills in insertion. This could be because fewer women take IUD, hence we are unable to get sufficient practice after a classroom training”.

Lack of adequate comprehensive counseling on contraceptive methods for clients was also reported by 58% of the providers. One provider stated, *“This is a high caseload facility, hence I am unable to provide comprehensive FP counseling to clients who come in”.* This finding is corroborated by another provider who noted that due to lack of comprehensive counseling by health care workers, some women who choose LARC are discouraged by their peers. *“I have noticed some clients who we inserted implants come back after about one month for removal. They say they have been advised by other women in the villages that it will harm them. Others say they have been having increased bleeding hence they don’t want the implants”.*

Infrastructural challenges were also mentioned by four providers who noted that their facilities lacked a room reserved for provision of FP services. “*The room currently used for FP services is also used for treatment of post abortion complications (PAC) and for examination of gender-based violence (GBV) victims, e.g., rape. Hence, when we receive PAC and GBV clients, the FP clients have to wait since theirs is not an urgent case”*, one of the providers reported.

All the providers interviewed mentioned lack of spousal support as a key barrier to uptake of LARC methods by women. “When you try to counsel women to take LARC methods, they fear them because they say their husbands normally palpate their arms to feel whether an implant has been inserted. For IUDs they do not like the fact that it is inserted through the vagina since the husband will feel it. They prefer depo (injectables) since their partners won’t know”, reported one provider.

## 4. Discussion

LARC methods may have a better chance at averting the unmet need for contraception in resource-limited settings [18] because they are more efficacious, provide better child spacing, are more cost-effective, and their effectiveness tends to be independent of user characteristics [19]. In addition, they play an important role in reducing contraceptive discontinuation. In this cross-sectional study among women of reproductive age seeking contraception services at public health facilities in rural Kenya, we assessed the method mix among clients initiating a contraceptive method and evaluated factors associated with uptake of LARC methods.

Our study found that among women who were interviewed, 20.6% opted for LARC and 79.4% chose short-term methods. The 2014 Kenya Demographic Health Survey findings, which was released in 2015 after our study was conducted, confirmed that the prevalence of LARC methods among modern contraceptive users (25.0%) were not as high as short-term methods (63.6%) in Kakamega County [5]. The slightly lower LARC uptake established in our study could be because the study was only conducted in public facilities; leaving out private sites, which serve 40% of FP clients [5]. The more recent findings of Performance Monitoring and Accountability (PMA) 2020 round 6 data (November–December 2017) for Kakamega County, indicate that whereas short-term methods are still more commonly used (48.8%) than LARC methods (41.0%) among clients using a contraceptive method, impressive strides have been made over the last 5 years [6]. The improvement in LARC uptake has largely been driven by an increase in the use of implants, which has steadily overtaken uptake of pills, but still lagging behind injectables [20].

In our study, tertiary education was an important predictor of LARC uptake. This is supported by other studies conducted in Ethiopia [11,21], Uganda [22], and the United States [7,23]. Increased level of education is associated with increased use of LARC methods in the current study. The most probable explanation is that more educated women have increased access to information on the benefits of LARC methods. However, some studies carried out in the United States [4] and in Ghana [24] had different results as they reported that uneducated women were more likely to use LARC methods than educated women. It is difficult to entirely dismiss or accept the influence of educational levels on LARC uptake due to the uneven distribution of education level in rural *vis-a-vis* urban settings, since in this study, 78% of the women resided in rural areas.

Additional findings showed that religion is associated with uptake of LARC methods as, in our study, being a Protestant reduced the odds of choosing a LARC method by almost half compared to Catholics. Our findings are further corroborated with analysis of data on contraceptive use from the Center for Disease Control’s Family Growth, which revealed that Catholics were more likely to use long-term FP methods than Protestants [25]. We posit that even though Catholic religion does not support contraceptive use, the congregants who decide to use are convinced of the importance of FP, hence they are more likely to choose more effective methods.

The current study also revealed that those who had no desire for more children were three times more likely to choose LARC methods compared to those who were undecided in regards to the number of children they wanted. This could be because LARC methods offer long-term protection against unwanted pregnancies and as such may be favored by women who do not want any more children. Kakamega County, just like all other counties in Kenya, has a lower uptake of permanent methods, including bilateral tubal ligation and vasectomy [5], among couples with a need to limit their fertility, hence women who are eligible for permanent methods could probably prefer LARC methods since they offer a longer-term protection. This is congruent to studies in Ghana [24], Ethiopia [26], and Pakistan [27] that have shown that there is high unmet need for permanent contraceptive methods, with women who are eligible opting to use LARC method instead. LARC methods are considered important alternatives to permanent methods especially in countries with lower rate of contraceptive use [28].

From the qualitative data, health system challenges, including staff shortage, inadequate skills, inadequate counseling, and inadequate infrastructure, where cited by health care providers as main barriers to provision of LARC methods. A key barrier for LARC uptake noted during the qualitative interviews was provider bias. While some health care workers openly mentioned being biased towards provision of short term method due to the longer time taken to counsel and insert an IUD or implant, a concept that is overlooked is that in the long run the workload will reduce because a client on LARC will make fewer visits to the clinic. This underscores the need to intensify mentorship of health care workers to appreciate the long term benefits that LARC confers on clients and on themselves. In addition, integrating cervical cancer screening while providing LARCs is an important aspect of holistic care but should not be used as a barrier to deny women access to contraceptive methods of their choice. Studies conducted in other regions have shown that education and training of health care workers on provision of LARC methods, including counseling, provider bias and integration of services, has been cited to be a significant predictor of LARC use [29,30,31]. These results suggest that addressing structural barriers hindering access to contraceptive services may increase uptake of LARC methods as availability of LARC methods should correlate with availability of trained personnel who do not act as barriers but instead are highly motivated to provide such methods [32,33]. The study further revealed that women opted for injectables due to interference and lack of support by their male partners hindering uptake of LARC. Other similar studies done in resource-limited settings in Uganda and Zambia also found that women prefer use of short-term methods in particular injectables because they can use these methods discretely [22,34,35]. Family planning programs need to continuously engage male partners to create an enabling environment that allows women to use their preferred method.

Our study had some limitations. The sample size was not representative of all women of reproductive age, as it only included women receiving a FP service at public health facilities; we intended to collect data from government health facilities where contraceptive commodities are provided free of charge. Public sector in Kenya provides FP services to 60% of women, by not including the private sector, our results might not be generalizable [6]. In addition, since the study was not population based, we could not estimate the prevalence of use of LARC among women of reproductive age.

As a cross-sectional study, the associations observed may not be causal. Specifically, the list of correlates we measured was likely not comprehensive, as comprehensive factors relating to the health care systems or the attitude of women on LARC were excluded. Including in-depth or focus group interviews with women may have enhanced our understanding of the reasons for women selecting their preferred method.

## 5. Conclusions

Our study concluded that LARC uptake is lower than short-term methods among women of reproductive-age in Kakamega County—a rural setting. Contraceptive method selection appeared to be influenced by socio-demographic (education, religion) and reproductive health (fertility intention) characteristics, although structural barriers were also noted to deter provision of LARC services at public health facilities. The results are critical for programming as they highlight the need for strategies that can strengthen LARC uptake by addressing health system factors including staff shortage, capacity building of health care workers on provision of LARC services, strengthening contraceptive counseling and male engagement in family planning program to create a supporting environment for contraceptive use. Future studies should assess whether the socio-demographic and reproductive health determinants have changed with the increased uptake of LARC methods among contraceptive users.

## Figures and Tables

**Table 1:** Socio-demographic and reproductive health characteristics of respondents.

Variable (*n* = 423)	Category	*n*	%
Age (years)	15–24	143	34
	25–34	187	44
	35+	93	22
Marital Status	Married	306	72
	Not Married	117	28
Education Level	None	11	3
	Primary	179	42
	Secondary	170	40
	Tertiary	63	15
Religion	None	5	1
	Muslim	40	9
	Catholic	121	29
	Protestant	257	61
Residence	Urban	92	22
	Rural	331	78
Occupation	Not Working	168	40
	Casual/Business	100	24
	Farming	126	30
	Paid Employment	29	7
Distance to facility M (SD)		2.5 (±2.3) km	
Ever given birth	Yes	392	93
	No	31	7
Sexual debut age (years)	≤19	292	69
	≥20	131	31
Parity	0	32	8
	1	109	26
	2–3	179	42
	4+	103	24
Desired Number of Children	1–2	118	28
	3–4	220	52
	5+	85	20
Fertility Intention	No more children	113	27
	Wants in 2 years	56	13
	Wants after 2 years	116	27
	Wants but unsure of timing	41	10
	Undecided	97	23
Age at first birth M (SD)		19.8 (±3)	

M (SD), mean (±standard deviation).

**Table 2:** Multivariable logistic regression of determinants of LARC uptake among women of reproductive age.

Variable	Category	LARC Uptake (*n* = 87)		Crude Odds Ratio (95% CI)	*p* Value	Adjusted Odds Ratio (95% CI)	*p* Value
		*n*	%				
Age (years)	15–24	17	11.9	Ref			
	25–34	43	23	2.21 (1.20–4.07)	**0.011**	0.84–3.15	0.148
	35+	27	29.1	3.03 (1.54–5.96)	**0.001**	0.88–4.20	0.103
Education Level	None/Primary	27	14.2	Ref			
	Secondary	41	24.1	1.92 (1.12–3.29)	**0.018**	0.98–3.16	0.061
	Tertiary	19	30.2	2.61 (1.33–5.12)	**0.005**	2.58 (1.10–6.03)	**0.029**
Religion	Catholic	34	28.1	Ref		Ref	
	None	1	20	0.64 (0.07–5.93)	0.694	0.52 (0.04–6.29)	0.605
	Muslim	10	25	0.85 (0.38–1.93)	0.703	0.73 (0.29–1.79)	0.489
	Protestant	42	16.3	0.51 (0.3–0.84)	**0.008**	0.42 (0.24–0.73)	**0.002**
Occupation	Not Working	24	14.3	Ref		Ref	
	Casual/Business	20	20	1.53 (0.78–2.88)	0.224	0.96 (0.45–2.05)	0.917
	Farming	34	27	2.22 (1.24–3.98)	**0.008**	1.62 (0.84–3.16)	0.152
	Paid Employment	9	31	2.71 (1.10–6.62)	**0.032**	1.29 (0.43–3.89)	0.651
Fertility Intention	Wants in 2 years	6	11.1	Ref		Ref	
	No more children	35	31.8	3.73 (1.46–9.55)	**0.006**	3.77 (1.37–10.42)	**0.001**
	Wants after 2 years	26	22.4	2.31 (0.89–6.00)	0.085	2.94 (1.07–8.08)	**0.037**
	Wants but unsure of timing	6	14.6	1.37 (0.41–4.61)	0.614	1.81 (0.51–6.37)	0.357
	Undecided	12	12.4	1.13 (0.40–3.20)	0.231	1.12 (0.37–3.25)	0.864
Age at first birth	≤19	24	14.0	Ref			
	≥20	63	25.1	2.07 (1.23–3.47)	**0.006**	1.66 (0.91–3.02)	0.098

Figures in bold are significant at *p* < 0.05.
